# Hybrid connectionist model determines CO_2_–oil swelling factor

**DOI:** 10.1007/s12182-018-0230-5

**Published:** 2018-04-26

**Authors:** Mohammad Ali Ahmadi, Sohrab Zendehboudi, Lesley A. James

**Affiliations:** Faculty of Engineering and Applied Science, Memorial University of Newfoundland, St. John’s, NL A1C 5S7 Canada

**Keywords:** CO_2_ injection, CO_2_ swelling, Genetic algorithm, Predictive model, Least-squares support vector machine

## Abstract

In-depth understanding of interactions between crude oil and CO_2_ provides insight into the CO_2_-based enhanced oil recovery (EOR) process design and simulation. When CO_2_ contacts crude oil, the dissolution process takes place. This phenomenon results in the oil swelling, which depends on the temperature, pressure, and composition of the oil. The residual oil saturation in a CO_2_-based EOR process is inversely proportional to the oil swelling factor. Hence, it is important to estimate this influential parameter with high precision. The current study suggests the predictive model based on the least-squares support vector machine (LS-SVM) to calculate the CO_2_–oil swelling factor. A genetic algorithm is used to optimize hyperparameters (*γ* and *σ*^2^) of the LS-SVM model. This model showed a high coefficient of determination (*R*^2^ = 0.9953) and a low value for the mean-squared error (MSE = 0.0003) based on the available experimental data while estimating the CO_2_–oil swelling factor. It was found that LS-SVM is a straightforward and accurate method to determine the CO_2_–oil swelling factor with negligible uncertainty. This method can be incorporated in commercial reservoir simulators to include the effect of the CO_2_–oil swelling factor when adequate experimental data are not available.

## Introduction

Due to the growing concern about global warming and the ongoing demand for energy resources, CO_2_-based enhanced oil recovery (EOR) methods have been attracting both the scientific and industrial interests. When CO_2_ is injected into depleted oil reservoirs, different mechanisms contribute to oil production (Farajzadeh et al. 2009; Godec et al. 2013; Kuznetsova and Kvamme 2002; Ma et al. 2016). These mechanisms depend on the operational conditions and oil composition. The most common oil production mechanisms in CO_2_-based EOR methods are oil viscosity reduction, oil swelling, condensation, vaporization, and interfacial tension (IFT) reduction (Abedini and Torabi 2014; Ahmadi et al. 2015; Bachu 2016; Czarnota et al. 2017; Farajzadeh et al. 2009; Li et al. 2013b, 2015; Shelton et al. 2016; Yang et al. 2012). Reducing the level of CO_2_ emissions in the atmosphere by the use of geological CO_2_ storage in depleted oil reservoirs as well as its role in the oil recovery processes highlights the importance of further studies of CO_2_ injection operations and the corresponding PVT behavior (Ahmadi et al. 2016a, b; Bachu 2016; Davis et al. 2010; Jamali and Ettehadtavakkol 2017; Kim and Santamarina 2014; Li et al. 2015; Li and Fan 2015; Liu and Wilcox 2012; Luo et al. 2013; Orr et al. 1982; Sell et al. 2012; Shelton et al. 2016; Yang et al. 2012; Yu et al. 2015; zeinali Hasanvand et al. 2013).

According to Rojas and Ali (1986) and Tunio et al. (2011), there are four effective mechanisms contributing to oil production using CO_2_-enhanced oil recovery strategies including (1) oil viscosity reduction, (2) oil swelling, (3) oil and water density reduction, and (4) vaporization and extraction of a portion of oil. It is clear that when CO_2_ is dissolved in the oil phase, the oil swells and its viscosity reduces. Hence, the variation in the swelling factor allows CO_2_ to substantially expand oil, which eventually improves the oil displacement and recovery (Perera et al. 2016). The immiscible CO_2_–EOR technique is dominated by the oil swelling phenomenon and oil viscosity reduction. The degree of oil swelling and oil viscosity change are dependent on different parameters including CO_2_ solubility in oil, pressure, temperature, and API degree of oil samples. CO_2_ solubility is generally considered as the most significant factor that influences the efficiency of CO_2_-based EOR techniques, particularly under low-pressure conditions. For instance, this mechanism was confirmed through implementation of pilot-scale tests in Turkey (Bagci 2007; Issever and Topkaya 1998; Perera et al. 2016).

Experimental investigations and numerical reservoir simulations on binary systems including hydrocarbons and CO_2_ were conducted to improve the hydrocarbon recovery (Bachu 2016; Bessières et al. 2001; Diep et al. 1998; Do and Pinczewski 1991; Fukai et al. 2016; Jamali and Ettehadtavakkol 2017; Kim and Santamarina 2014; Kiran et al. 1996; Kwak and Kim 2017; Li et al. 2013a, 2015; Li and Fan 2015; Luo et al. 2007, 2013; Lv et al. 2015; Mulliken and Sandler 1980; Shelton et al. 2016; Yang and Gu 2005). Most of these studies investigated the oil swelling effect primarily as a result of CO_2_ dissolution in the light fractions of oil. Bessières et al. (2001) and Kiran et al. (1996) examined the variation in the volume of several CO_2_–alkane systems. They concluded that the excess volume follows a sigmoidal change with the composition/concentration of CO_2_. The oil swelling effect was determined by the volume swelling coefficient defined by Yang and Gu (2005) and Yang et al. (2012). These investigations reveal that with an increase in the pressure (and consequently the solubility of CO_2_ in oil), the volume swelling coefficient of the oil phase increases. Yang et al. (2012) studied the behavior of oil swelling through qualitative analysis of the dispersion of CO_2_ in oil. Experiments at reservoir conditions (high temperature, high pressure, and live oil composition) are, however, challenging. A swelling/extraction experiment is a well-known technique to record composition and reservoir fluid volume changes due to CO_2_ dissolution in reservoir oil at a given temperature. Swelling experiments are typically carried out in a high-pressure-resistant visual PVT cell with a constant volume, which is first filled with a specific volume of dead or stock-tank oil (Tsau et al. 2010). Depending on the number of steps required to reach the desired pressure, CO_2_ is injected gradually to achieve a proper pressure increase. The main assumption of the swelling experiment is neglecting the vaporization of intermediate components of oil into the CO_2_ phase until reaching the minimum miscibility pressure. The oil volume change owing to the swelling effect at each pressure step is recorded and the amount of CO_2_ dissolved in the oil is measured. An increase in the pressure results in vaporization of a part of oil components, and the oil-rich phase shrinks. It should be noted that the phase behavior of the CO_2_ and oil system can be visually detected using a swelling test. Various parameters including the bubble point pressure, solubility of CO_2_, and swelling factor are usually employed to tune the equation of state (EOS) for the phase behavior modeling purposes (Tsau et al. 2010). Different sizes of visual PVT cells can be utilized for swelling experiments; these cells include 140 mL (Hand and Pinczewski 1990), 170 mL (Harmon and Grigg 1988), 190 mL (Orr et al. 1981), and 450 mL. Holm and Josendal (1982) recommended that a volume of 30% of the cell volume should be considered as the sample size for the swelling test. Therefore, the proper volume range is 40–100 mL of the crude oil sample to perform the swelling tests using the corresponding PVT cells. The most important issue with the sampling size is the time needed to achieve an equilibrium condition after each pressure change. The mixing process of large volumes of gas and oil at a given pressure seems to be another major concern in such a swelling test.

Thomas and Monger-McClure (1991) studied the effect of the CO_2_–oil swelling factor on oil recovery from light oil reservoirs using cyclic CO_2_ injection. They correlated the oil incremental value to the CO_2_–oil swelling factor. Based on the results, an increase in the CO_2_–oil swelling factor led to an increase in the amount of produced oil (Thomas and Monger-McClure 1991).

Dong et al. (2001) determined the CO_2_–oil swelling factor by comparing the measured densities of the dead oil sample, reservoir live oil, and mixture of CO_2_ and reservoir oil. Ghedan (2009) claimed that at high CO_2_ concentrations the CO_2_–oil swelling factor will be 1.25–1.6; in most of the cases, the CO_2_ content should be greater than 50%. Ning et al. (2011) carried out several multiple contact experiments (MCEs) to figure out the contribution of oil swelling as well as reduction in oil viscosity to the oil production from Alaska North Slope viscous oil. Heidaryan and Moghadasi (2012) investigated the influence of swelling and viscosity reduction on oil production using both experimental and theoretical methods. Based on their research outcome, they concluded that the optimum value of the CO_2_–oil swelling factor should be 1.7 to reach the maximum oil production from the reservoir (Heidaryan and Moghadasi 2012).

Through a systematic research work, Sugai et al. (2014) experimentally determined oil swelling factors in porous media using two different types of micromodels (e.g., fine beads and coarse beads). They investigated the effect of interfacial area on the oil swelling and CO_2_–oil swelling factors. They used a digital camera to take images to determine the amount of oil trapped in the micromodels at different times. They obtained the swelling factor from the tests after a constant saturation degree in the porous systems was confirmed. In addition, they employed an oil–CO_2_ simple contact model in a visual cell to determine CO_2_–oil swelling factors at different pressures via utilizing a digital camera and an image processing method. They compared CO_2_–oil swelling factors from both types of the experiments to decide what other parameters should be taken into account to further improve the accuracy and reliability of the existing approach. According to the experimental results, they concluded that an increase in the interfacial area results in increasing the oil swelling. In other words, the swelling factor in the case of the fine bead micromodel was larger than that in the coarse bead micromodel due to an increase in the interfacial area (Sugai et al. 2014). Or et al. (2016) experimentally investigated the contribution of CO_2_–oil swelling and viscosity reduction to the oil recovery through implementation of CO_2_ gas foaming in heavy oil reservoirs. It was concluded that CO_2_ foam swelling increases with an increase in the pressure drawdown in a well. Also, further swelling of foamy oil can mobilize the residual oil towards the producer well, especially in the immobilized zone (Or et al. 2016).

Habibi et al. (2017) carried out experiments on CO_2_–oil systems to determine the interaction between CO_2_ and oil in tight rock samples. They conducted constant composition experiments (CCEs) to determine the CO_2_–oil swelling factor and other measurable fluid and thermodynamic characteristics. Also, they performed CO_2_ cyclic injection experiments to determine the amount of oil recovery. The CO_2_-oil swelling factor in their study was defined as “the volume of the oil after CO_2_ injection divided by the volume of the oil before CO_2_ injection into the cell.” In their experiments, increasing CO_2_ concentration from 48.4% to 71.1% resulted in an increase in the CO_2_–oil swelling factor from 1.21 to 1.39, respectively. According to their experimental data, the oil swelling and expansion, CO_2_ dissolution into the oil, and CO_2_ diffusion into core samples are the main mechanisms contributing to the oil production (Habibi et al. 2017).

There are a few studies that have developed a reliable correlation or a deterministic model for predicting CO_2_–oil swelling factors. Welker (1963) proposed a very simple correlation to estimate the CO_2_–oil swelling factor. Their correlation suffers from the lack of applicability, particularly for light and intermediate crude oil samples. Simon and Graue (1965) developed a graphical method to determine the oil swelling factor. Their method was developed based on limited data samples from heavy crudes. Chung et al. (1988) proposed a simple correlation to estimate the oil swelling factor for CO_2_/heavy crude oil systems. Emera and Sarma (2006) developed a correlation to forecast the oil swelling factor for both light and heavy crude oils. However, they utilized a limited number of data points while developing their correlation. Table 1 demonstrates a summary of correlations and models to calculate the CO_2_–oil swelling factor.

**Table 1:** Correlations and models for calculating CO_2_–oil swelling factor (SF)

Correlation	Considerations/limitations	References
$SF = 1.0 + \frac{{0.35\left( {{\text{solubility}} \left( {\text{scf/bbl}} \right)} \right)}}{1000}$	Developed for oils at *T* = 80 °F and 20° API < oil gravity < 40° API	Welker (1963)
Graphical correlation: This model is a function of CO_2_ solubility, oil molecular weight (*MW*), and oil density at 60 °F. Not recommended for high-pressure ranges	*P* < 2300 psi 110 °F < *T* < 250 °F 12° API < oil gravity < 33° API	Simon and Graue (1965)
$SF = \frac{{\rho_{\text{l}} }}{\rho - S}$ *S* = CO_2_ solubility, g/cm^3^ *ρ* = oil density without CO_2_ at the same temperature and 1 atm pressure, g/cm^3^ *ρ*_l_ = solution density, g/cm^3^	16.89° API oil gravity 75 °F < *T* < 200 °F 14.7 psi < *P* < 5014.7 psi	Chung et al. (1988)
For *MW* > 300 $SF = 1 + 0.3302Y - 0.8417Y^{2} + 1.5804Y^{3} - 1.074Y^{4} - 0.0318Y^{5} + 0.21755Y^{6}$ For *MW* < 300 $SF = 1 + 0.48411Y - 0.9928Y^{2} + 1.6019Y^{3} - 1.2773Y^{4} + 0.48267Y^{5} - 0.06671Y^{6}$ $Y = 1000 \times \left( {\left( {\left( {\frac{\gamma } {MW}} \right) \times {\text{sol}}({\text{mole}}\;{\text{fraction}})^{2} } \right)^{{\exp \left( {\frac{\gamma } {MW}} \right)}} } \right)$	23 °C < *T* < 121.1 °C 0.1 MPa < *P* < 27.4 MPa 12° API < oil gravity < 37° API	Emera and Sarma (2006)

Vapnik (1998) proposed the support vector machine (SVM) as an extended version of conventional artificial intelligent tools. SVM is a practical method which has been widely used for classification, regression, and pattern recognition (Cortes and Vapnik 1995). The principle idea of SVM is to transform the nonlinear input space to a higher-dimensional feature space to find a hyperplane via nonlinear mapping (Baylar et al. 2009; Cortes and Vapnik 1995). It is based on the statistical learning theory (SLT) and structural risk minimization (SRM) concepts (Mehdizadeh and Movagharnejad 2011). SVM tools obtain the solution via solving the quadratic programming (QP); the SVM always results in a global optimum solution, unlike other regression techniques such as neural networks, as the QP problem is a convex function (Vong et al. 2006). However, it suffers from computational burden.

The LS-SVM has not been used to model the CO_2_–oil swelling factor in the literature, to the best of our knowledge. This study employs the least-squares support vector machine (LS-SVM) paradigm, as a hybridized version of the original SVM method, to calculate the CO_2_–oil swelling factor. A genetic algorithm (GA) is utilized as an optimization technique to optimize the hyperparameters of the LS-SVM model. Through the comprehensive literature review, extensive experimental data are used for model development and validation.

## Theory

### Least-squares support vector machine (LS-SVM)

Suykens and Vandewalle (1999) proposed the least-squares support vector machine (LS-SVM) model as an alternate formulation of the SVM regression. The LS-SVM enjoys similar advantages as SVM. Also, it requires solving only a set of linear equations instead of a quadratic programming (QP) problem, which is computationally less demanding.

Given the training set $\{ x_{k} ,y_{k} \}$, $k = 1, 2, \ldots ,N$, where $x_{k} \in {\mathbb{R}}^{n}$ is the *k*th input data in the input space and $y_{k} \in {\mathbb{R}}$ represent the output variable for the given input variable (i.e., $x_{k}$) and $N$ refers to the number of the training samples. Using a nonlinear function $\varphi ( \cdot )$, which maps the training set in the input space to a high (and possibly infinite)-dimensional space, the following regression model is constructed:

1 $$ y = \omega^{\text{T}} \varphi (x) + b\quad {\text{with}}\quad \omega \in {\mathbb{R}}^{n} ,\quad b \in {\mathbb{R}},\quad \varphi ( \cdot ) \in {\mathbb{R}}^{n} \to {\mathbb{R}}^{{n_{h} }} ,\quad n_{h} \to \infty $$

in which, $\omega$ denotes the weight vector and $b$ signifies a bias term. Note that the superscript “*n*” refers to the dimension of data space and “$n_{h}$” is attributed to the higher-dimensional feature space (Vong et al. 2006). When the LS-SVM is applied, a new optimization case will be generated. The implemented strategy deals with the following optimization problem:

2 $$ \frac{\hbox{min} }{\omega ,b,e}\quad {\mathcal{J}}\left( {\omega ,e} \right) = \frac{1}{2}\omega^{\text{T}} \omega + \frac{1}{2}\gamma \mathop \sum \limits_{k = 1}^{N} e_{k}^{2} $$

subject to the following equality constraint:

3 $$ y_{k} = \omega^{\text{T}} \varphi \left( {x_{k} } \right) + b + e_{k} \quad k = 1, 2, \ldots , N $$

where $\gamma$ represents the regularization parameter, which compromises between the model’s complexity and the training error (Mehdizadeh and Movagharnejad 2011), and $e_{k}$ is the regression error. The Lagrangian is constructed as follows in order to find the solution of the un-constrained optimization problem:

4 $$ {\mathcal{L}}\left( {\omega ,b,e,\alpha } \right) = {\mathcal{J}}\left( {\omega ,e} \right) - \mathop \sum \limits_{k = 1}^{N} \alpha_{k} \left\{ {\omega^{\text{T}} \phi \left( {x_{k} } \right) + b + e_{k} - y_{k} } \right\} $$

where $\alpha_{k}$ stands for the Lagrange multiplier or support value. To attain the solution of Eq. (4), differentiating the equation with respect to $\omega ,b,e_{k} ,\alpha_{k}$ gives:

5 $$ \frac{{\partial {\mathcal{L}}\left( {\omega ,b,e,\alpha } \right)}}{\partial \omega } = 0 \to \omega = \mathop \sum \limits_{k = 1}^{N} \alpha_{k} \varphi \left( {x_{k} } \right) $$

6 $$ \frac{{\partial {\mathcal{L}}\left( {\omega ,b,e,\alpha } \right)}}{\partial b} = 0 \to \mathop \sum \limits_{k = 1}^{N} \alpha_{k} = 0 $$

7 $$ \frac{{\partial {\mathcal{L}}\left( {\omega ,b,e,\alpha } \right)}}{{\partial e_{k} }} = 0 \to \alpha_{k} = \gamma e_{k} ,\quad k = 1, \ldots , N $$

8 $$ \frac{{\partial {\mathcal{L}}\left( {\omega ,b,e,\alpha } \right)}}{{\partial \alpha_{k} }} = 0 \to y_{k} = \varphi \left( {x_{k} } \right)\omega^{\text{T}} + b + e_{k} ,\quad k = 1, 2, \ldots ,N $$

After removing the variables $\omega$ and $e$, one acquires the Karush–Kuhn–Tucker system as follows:

9 $$ \left[ {\begin{array}{*{20}c} 0 & {1_{\upsilon }^{\text{T}} } \\ {1_{\upsilon } } & {\varOmega + \gamma^{ - 1} I} \\ \end{array} } \right]\left[ {\begin{array}{*{20}c} b \\ \alpha \\ \end{array} } \right] = \left[ {\begin{array}{*{20}c} 0 \\ y \\ \end{array} } \right] $$

In Eq. (9), $y = \left[ {y_{1} \ldots y_{N} } \right]^{\text{T}}$, $1_{N} = \left[ {1 \ldots  1} \right]^{\text{T}}$, $\alpha = \left[ {\alpha_{1} \ldots  \alpha_{N} } \right]^{\text{T}}$, *I* is an identity matrix, and $\varOmega_{kl} = \varphi \left( {x_{k} } \right)^{\text{T}} \cdot \varphi \left( {x_{l} } \right) = K\left( {x_{k} ,x_{l} } \right)\forall k,\quad l = 1, 2, \ldots ,N$. $K\left( {x_{k} ,x_{l} } \right)$ is the kernel function and must meet Mercer’s condition (Li et al. 2008). Three typical choices for the kernel function are:

- $$ K\left( {x,x_{k} } \right) = x_{k}^{\text{T}} x $$
- $$ K\left( {x,x_{k} } \right) = (\tau + x_{k}^{\text{T}} x)^{d} $$
- $$ K\left( {x,x_{k} } \right) = \exp \left( {{{ - x - x_{k}^{2} } \mathord{\left/ {\vphantom {{ - x - x_{k}^{2} } {\sigma^{2} }}} \right. \kern-0pt} {\sigma^{2} }}} \right) $$

The resulting formulation of LS-SVM model for function estimation becomes:

10 $$ y\left( x \right) = \mathop \sum \limits_{k = 1}^{N} \alpha_{k} K\left( {x,x_{k} } \right) + b $$

where $\tau$ refers to the slope, *d* stands for the polynomial degree, *σ*^2^ is the kernel sample variance, and $\left( {b, \alpha } \right)$ represents the solution to the linear system of equations shown in Eq. (9).

In the literature, some comprehensive descriptions of the SVM are available (Burges 1998; Suykens and Vandewalle 1999; Vapnik 1998). The theory of LS-SVM is systematically explained by a number of researchers (Suykens and Vandewalle 1999; Suykens et al. 2002). Also, Liu et al. (2005a, b, 2007) provide a detailed comparison of the SVM and LS-SVM methods.

### Genetic algorithm

Genetic algorithm (GA) is a stochastic method to solve optimization problems involving a fitness criterion, survival of the fittest, and different genetic operators, including crossover and mutation to satisfy a pre-defined fitness quantity, resembling the Darwinian evolution by natural selection (Niazi et al. 2008). The significant feature of the GAs and the other similar evolutionary algorithms is that they are derivative-free. The stochastic nature of the algorithm with dynamic evaluation of the fitness function brings a powerful systematic random search engine. This approach is an alternative to derivative-based methods to deal with problems in which the fitness function is non-differentiable, discontinuous, highly nonlinear, with multiple local optima, or stochastic (Reihanian et al. 2011).

## Data gathering

Extensive data points for the CO_2_–oil swelling factor have been extracted from the literature (Abedini et al. 2014; Chung et al. 1988; Mosavat et al. 2014; Tsau et al. 2010; Wei et al. 2017). The statistical parameters for these data samples are reported in Table 2. As it is clear from Table 2, the data samples contain a broad range of crude oils from heavy oils to extra-light oil samples. The collected data also cover a wide range of temperature, pressure, and CO_2_ solubility.

**Table 2:** Statistical parameters of the data points (Abedini et al. 2014; Chung et al. 1988; Mosavat et al. 2014; Tsau et al. 2010; Wei et al. 2017) used for developing LS-SVM model

Parameter	Minimum	Maximum	Average
Oil gravity, API degrees	16.8	46.11	32.8
Temperature *T*, °F	68	200	109.5
Pressure *P*, psi	14.7	4100	1187.6
CO_2_ concentration (mole fraction)	0	0.86	0.525

## Methodology

In this paper, four parameters are considered as input variables to the LS-SVM model. These parameters are (1) CO_2_ concentration in oil (mole fraction of CO_2_), (2) pressure, (3) temperature, and (4) the oil API degree. The output variable from the LS-SVM model is the CO_2_–oil swelling factor.

A total number of 225 data samples were extracted from the literature to develop our LS-SVM model to estimate the CO_2_–oil swelling factor. The data samples were divided into two data sets. The first set (also called the training data series) contained 80% of the total data points to construct the LS-SVM model. The second set of data contained 20% of the entire data points employed to validate the LS-SVM model.

The radial basis function (RBF) was selected because of its promising performance and simplicity as it only contains one adjustable parameter and has been successfully applied (Ahmadi 2015; Keerthi and Lin 2003; Reihanian et al. 2011). In the model development using LS-SVM with the RBF kernel function, according to Eqs. (9) and (10), the optimization of *γ* and *σ*^2^ is a crucial task. It was confirmed that the optimal magnitudes of these two vital parameters are required to better design a LS-SVM model towards greater precision and generalization (Vong et al. 2006).

**Fig. 1:**
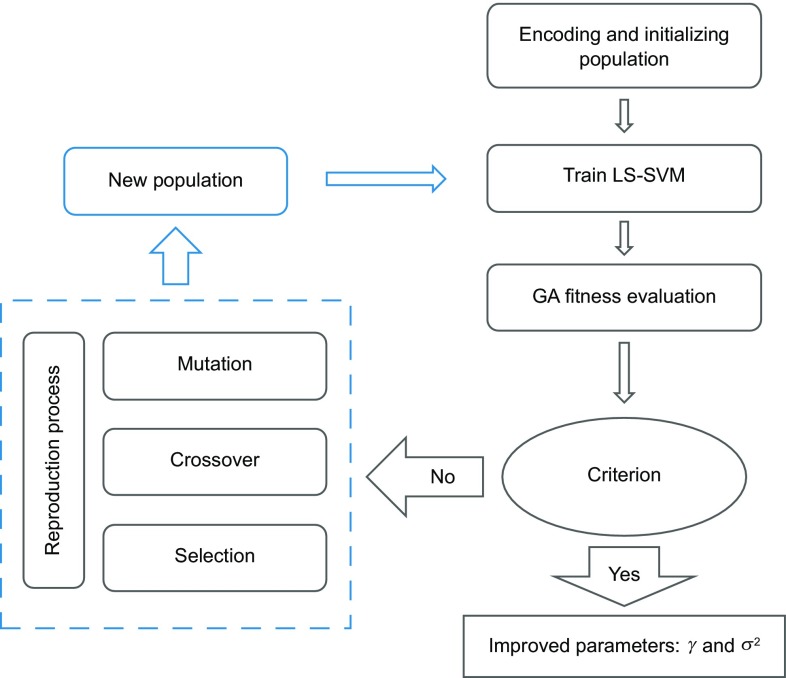
Flowchart of hyperparameters selection based on GA

According to Ahmadi and Ebadi (2014), Ahmadi et al. (2014a, b), and Fazeli et al. (2013), the application of non-population-based optimization methods such as simulated annealing and Levenberg–Marquardt (LM) is not recommended due to their drawback in handling the nonlinearity in SVM methods. GA was applied in this research study to optimize the parameters of LS-SVM (*γ* and *σ*^2^) and the average absolute relative deviation (AARD). The flowchart for the hyperparameter optimization using a GA algorithm is depicted in Fig. 1. The optimization procedure was repeated several times to attain the most plausible solution corresponding to the global optimum of the fitness function. As a result, values of *σ*^2^ and *γ* were obtained: 0.268829 and 33.4091, respectively.

## Results and discussion

**Fig. 2:**
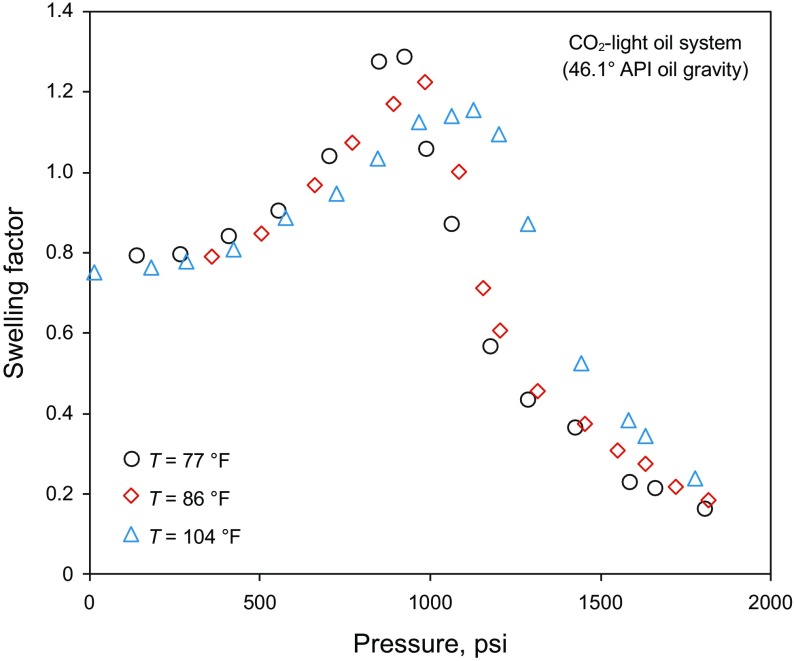
Swelling factor of CO_2_–light oil system versus pressure at various temperatures (Abedini et al. 2014; Chung et al. 1988; Mosavat et al. 2014; Tsau et al. 2010; Wei et al. 2017), light oil with an API gravity of 46.11°

**Fig. 3:**
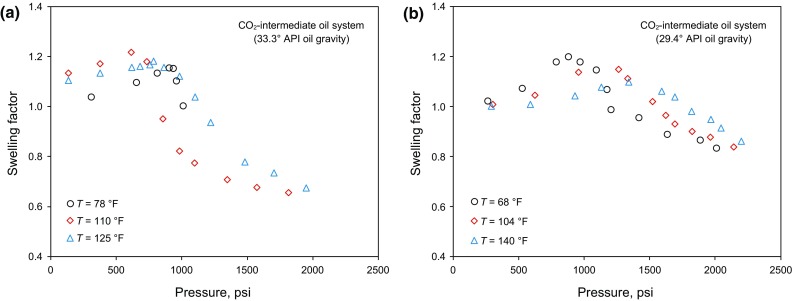
Variations of swelling factor of CO_2_–intermediate oil system with pressure and temperature (Abedini et al. 2014; Chung et al. 1988; Mosavat et al. 2014; Tsau et al. 2010; Wei et al. 2017): **a** An oil with an API gravity of 33.3° and **b** An oil with an API gravity of 29.4°

**Fig. 4:**
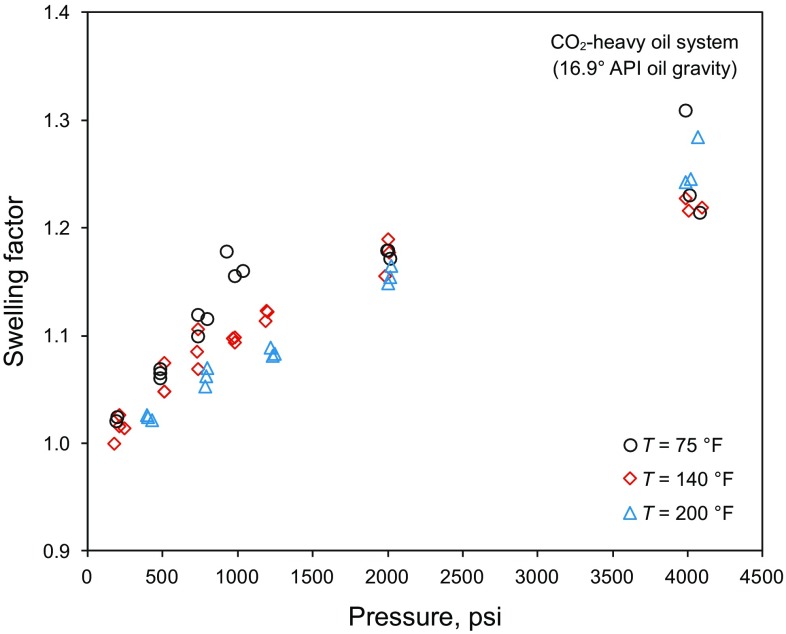
Swelling factor of CO_2_–heavy oil system versus pressure at different temperatures (Abedini et al. 2014; Chung et al. 1988; Mosavat et al. 2014; Tsau et al. 2010; Wei et al. 2017), heavy oil with an API gravity of 16.9°

This study presents a new deterministic approach to obtain the swelling factor with higher accuracy. The oil swelling factor for the system of CO_2_ and light oil versus pressure at different temperatures is demonstrated in Fig. 2. The variations of the oil swelling factor with pressure at various temperatures are shown in Figs. 3 and 4 for intermediate and heavy oil samples, respectively.

Mean-squared error (MSE) and coefficient of determination (*R*^2^) are employed in this statistical analysis as the performance evaluation criteria for the LS-SVM model in estimating the CO_2_–oil swelling factor. The expressions to obtain MSE and *R*^2^ are given below:

11 $$ {\text{MSE}} = \frac{1}{N}\mathop \sum \limits_{i = 1}^{N} \left( {y_{i}^{\text{actual}} - y_{i}^{\text{predicted}} } \right)^{2} $$

12 $$ R^{2} = 1 - \frac{{\mathop \sum \nolimits_{i = 1}^{N} \left( {y_{i}^{\text{actual}} - y_{i}^{\text{predicted}} } \right)^{2} }}{{\mathop \sum \nolimits_{i = 1}^{N} \left( {y_{i}^{\text{actual}} - \overline{{y^{\text{actual}} }} } \right)^{2} }} $$

where *N* represents the number of data points, $y_{i}^{\text{actual}}$ denotes the *i*th observation (real data), $y^{\text{predicted}}_{i}$ is the *i*th output from the model, and $\overline{{y^{\text{actual}} }}$ signifies the average magnitudes of observations. The values of MSE and *R*^2^ are tabulated in Table 3 for training, testing, and overall data stages. The GA-LS-SVM predictions are satisfactory if *R*^2^ and MSE are close to 1 and 0, respectively. As can be seen in Table 3, these criteria were fulfilled.

**Table 3:** Performance of GA-LS-SVM method with optimized parameters for prediction of swelling factor in terms of statistical parameters

Statistical parameters	Training data	Testing data	Overall data
MSE	0.00016	0.0009	0.0003
*R* ^2^	0.9944	0.9931	0.9953
Average absolute relative deviation (AARD)^a^	0.7918	4.549	1.5433
Maximum absolute error (MAE)	5.3403	5.4205	5.4205

^a^ $$ {\text{AARD}} = \frac{{\sum \left| {\frac{{y^{\text{actual}} - y^{\text{predicted}} }}{{y^{\text{actual}} }}} \right|}}{n} $$

**Fig. 5:**
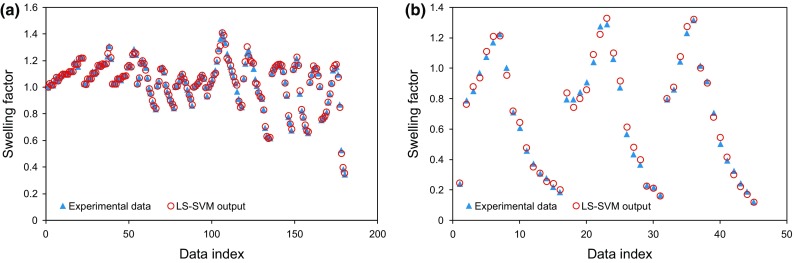
Comparison between estimated and measured swelling factors versus data index: **a** Training data and **b** Testing data

Figure 5 depicts a comparison between the experimental data for the CO_2_–oil swelling factor and the values estimated by the LS-SVM. Figure 5a shows a comparison between the estimated and experimental data in the training phase. Figure 5b demonstrates a comparison between the actual and predicted CO_2_–oil swelling factor behavior against the data index in the testing phase. As illustrated in Fig. 5, there is an excellent match between the oil swelling factor estimated from the LS-SVM method and those from experiments.

**Fig. 6:**
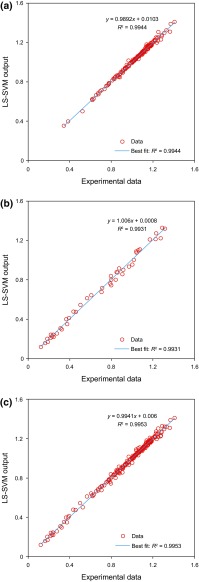
Scatter plot of estimated and measured swelling factors: **a** Training data; **b** Testing data; and **c** All data

Figure 6 illustrates the regression plot between the CO_2_–oil swelling factor determined by LS-SVM model and the experimental data points. Figure 6a depicts the scatter plot for results obtained in the training phase of the LS-SVM model. As shown in Fig. 6a, the linear fit to data *y* = 0.9892*x* + 0.0103 has a high correlation of coefficient (*R*^2^ = 0.9944), meaning that the training phase of the LS-SVM model is performed very well. The results achieved over the testing (validation) phase are displayed in the form of a scatter plot in Fig. 6b, based on the developed LS-SVM tool. As depicted in Fig. 6b, the high value of the correlation coefficient (*R*^2^ = 0.9931) between the predicted and experimental oil swelling factor shows the superior performance of the LS-SVM model. Figure 6c illustrates the regression plot for the whole data set. The predicted swelling factor values are found to be scattered around the *y* = *x* line, indicating that the LS-SVM model that is optimized by GA predicts the swelling factor very well.

**Fig. 7:**
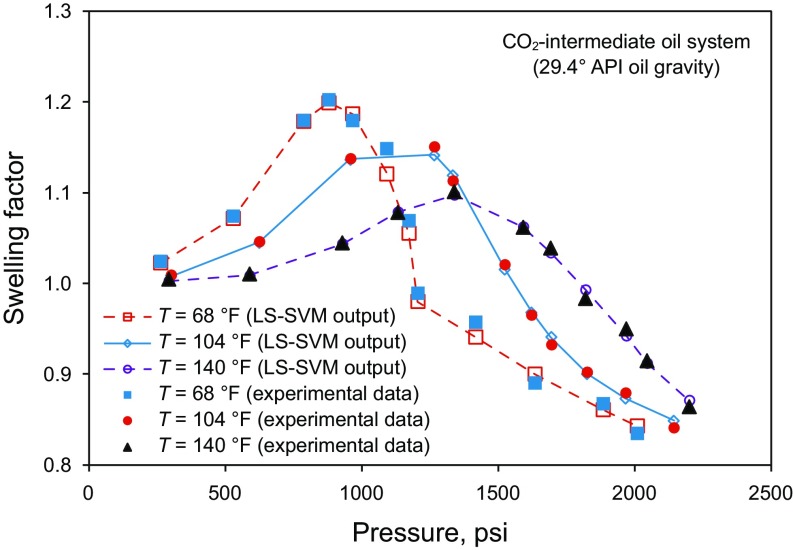
Comparison between calculated and measured swelling factors versus pressure at different temperatures for the CO_2_–intermediate oil system (The oil API gravity is 29.4°)

Figure 7 represents a comparison between the CO_2_–oil swelling factor determined by the LS-SVM model and the real data versus pressure at different temperatures. As shown in Fig. 7, the LS-SVM model follows the trend of experimental data points for an immediate oil of 29.4° API gravity. As the experimental data points show, at a constant pressure, the magnitude of swelling factor lowers with increasing the temperature. This behavior was confirmed by the LS-SVM model. This implies that the proposed LS-SVM model for determination of CO_2_–oil swelling factor is valid and acceptable in terms of technical and conceptual prospects.

**Fig. 8:**
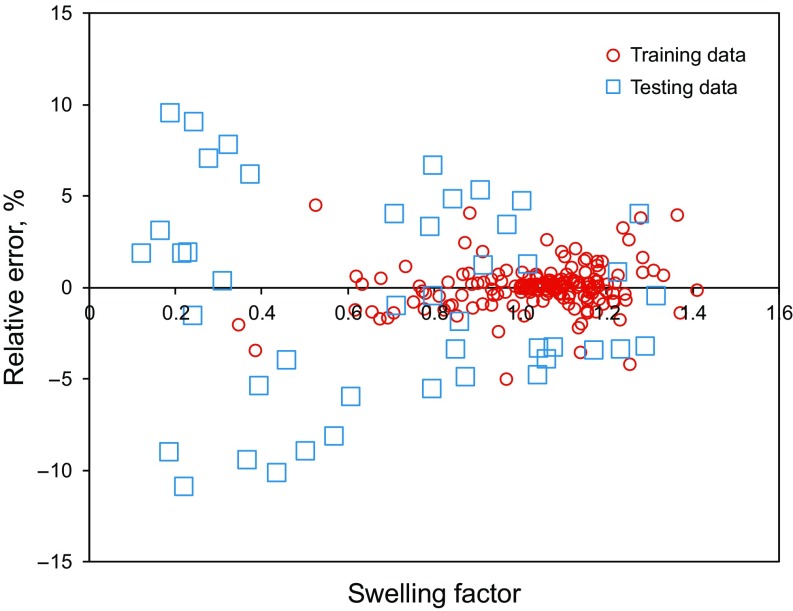
Relative error distribution of the estimated target variable (e.g., swelling factor)

Figure 8 shows the relative error distribution for both the training and testing phases in developing the LS-SVM model. According to Fig. 8, the maximum relative error between the outputs of the LS-SVM model and the experimental CO_2_–oil swelling factors is within ± 5% for the training phase. Also, the maximum relative error between the CO_2_–oil swelling factor calculated by the LS-SVM model and experimental data points is within ± 15% for the testing phase.

**Fig. 9:**
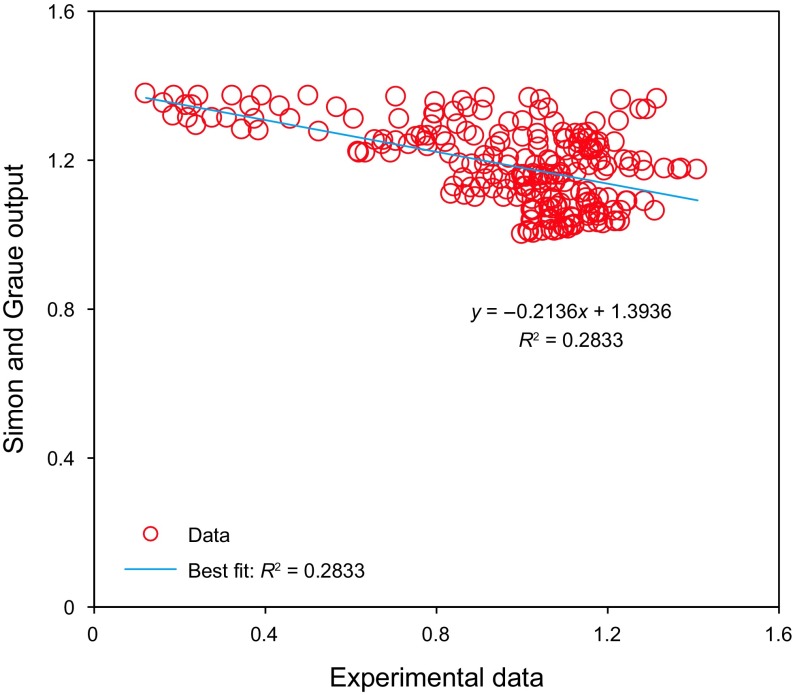
The Simon–Graue (1965) outputs versus measured swelling factors

Simon and Graue (1965) proposed a graphical method for determination of the CO_2_–oil swelling factor. In this method, the minimum value of the CO_2_–oil swelling factor is equal to 1 and the maximum value is equal to 1.38. Also, the Simon and Graue technique offers acceptable values for swelling factor within the limited ranges of API, temperature, and CO_2_ solubility (Table 1). Hence, this graphical method is not able to provide reliable outputs over wide ranges of the input parameters. Figure 9 demonstrates the scatter plot of the results obtained by the graphical method proposed by Simon and Graue (1965) versus the experimental values of the CO_2_–oil swelling factor. As it is clear from Fig. 9, the linear fit line has a low correlation coefficient (*R*^2^). Also, the linear fit has a negative slope, concluding that the value of oil swelling factor at the lower boundary is overestimated.

**Fig. 10:**
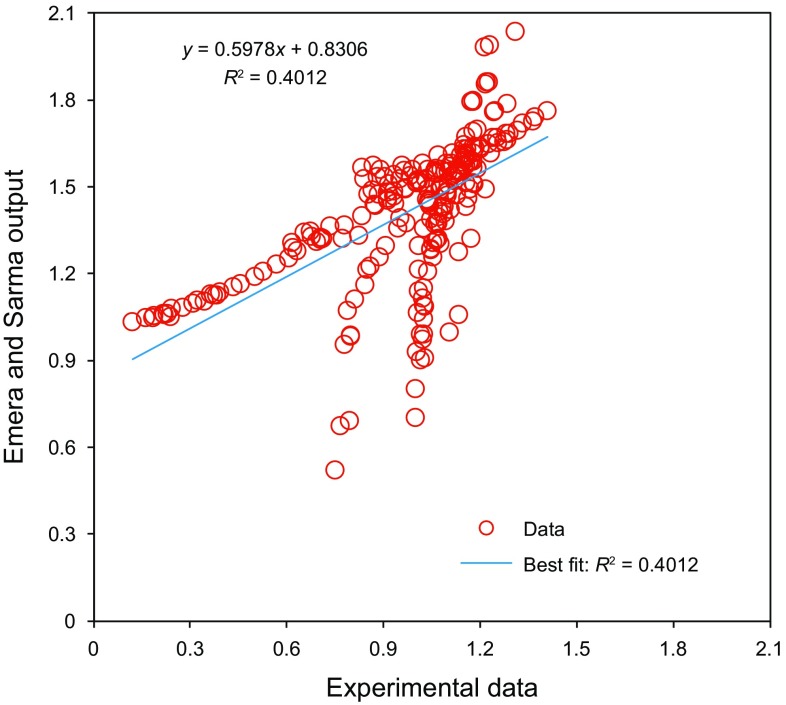
Scatter plot of values of the target parameter estimated by Emera and Sarma (2006) correlation and measured swelling factors

Figure 10 presents a comparison between the objective function values calculated by Emera and Sarma (2006) correlation and the real data of the CO_2_–oil swelling factor. Based on Figs. 9 and 10, the linear fit of the data obtained by Emera and Sarma (2006) correlation has a higher value of correlation of coefficient in comparison with the method proposed by Simon and Graue (1965). This is because the correlation introduced by Emera and Sarma (2006) was developed using a wider range of data points. However, this correlation still suffers from the common drawback for the most empirical correlations which can offer reliable outputs within limited ranges of input parameters (Table 1). As illustrated in Fig. 10, the Emera and Sarma (2006) correlation underestimates the magnitudes of the swelling factor in the middle range of the data.

Table 4 reports the maximum absolute error (MAE) and the average absolute relative deviation (AARD) for three different models based on the experimental data available for the CO_2_–oil swelling factor. The MAE of the LS-SVM model is lower, compared to the Emera and Sarma (2006) and Simon and Graue (1965) methods. This superior performance is attributed to the high predictive capability of the developed tool, proper procedure for the training phase, and careful selection of data samples. Using a broader range of data samples enables us to develop a more precise and reliable technique to calculate the CO_2_–oil swelling factor.

**Table 4:** Maximum absolute error and average absolute relative deviation to indicate the difference between the predicted values and experimental data

Method	Maximum absolute error	Average absolute relative deviation
LS-SVM model	5.42	1.5433
Emera and Sarma method	91.2154	66.92
Simon and Graue method	125.70	56.87

It should be noted the correlation proposed by Emera and Sarma (2006) is currently being used in the Computer Modelling Group (CMG) reservoir simulator package. It suggests that the LS-SVM strategy introduced in this research work can be included in the commercial reservoir simulators for various applications such as simulation of gas injection processes in the petroleum industry.

**Fig. 11:**
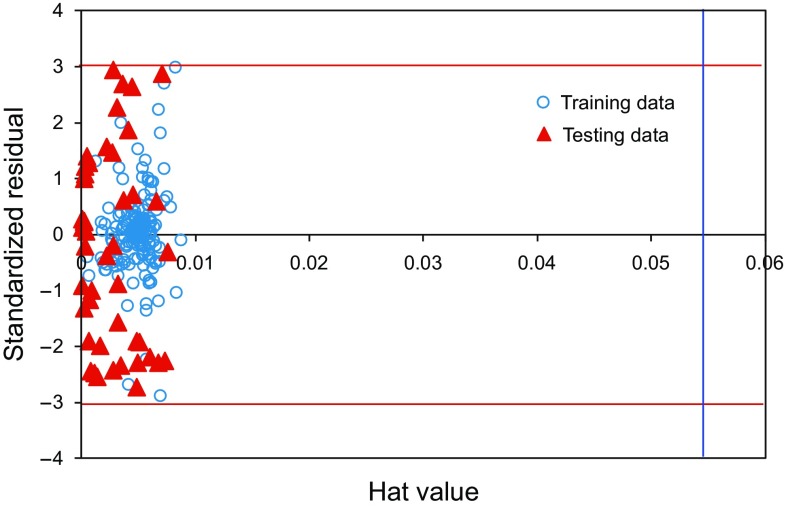
Detection of the possible doubtful measured objective function and the applicability domain of the suggested approach for the CO_2_–oil swelling factor (The hat value (*H*) is 0.0555)

Appropriate statistical methods for identifying the applicability of the model are required for outlier detection. Recognition of outliers is to determine which data points may differ from the bulk of the data present in the data bank under study (Gramatica 2007; Rousseeuw and Leroy 2005). For examining the capability of the LS-SVM model, the approach of Leverage Value Statistics has been carried out (Goodall 1993; Gramatica 2007). A graphical method (William plot) is used for outlier determination in this research work. The William plot depicts the standardized residual of the outputs versus corresponding hat (*H*) values. Further details on the mathematical backgrounds and computational procedure of the William method can be found in the literature (Goodall 1993; Gramatica 2007; Rousseeuw and Leroy 2005). Figure 11 represents the William plot for the results obtained from the LS-SVM model while estimating the CO_2_–oil swelling factor. Having the majority of data points in the ranges of $0 \le H \le 0.055$ and $- 3 \le R \le 3$ reveals that the LS-SVM model is convincing and reliable in terms of statistical criteria. In addition, it conveys the message that the entire data are located within the acceptable domains, again confirming the LS-SVM model offers accurate and satisfactory predictions.

**Fig. 12:**
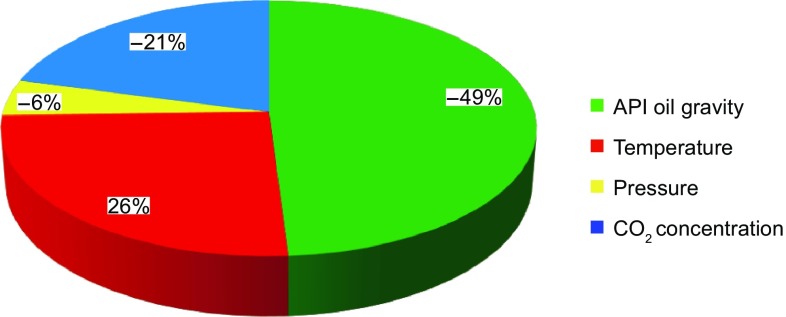
Relative importance of the independent variables affecting the swelling factor

Analysis of variance (ANOVA) was used to determine the relative importance of all the input parameters which are incorporated in this modeling strategy to develop the connectionist tool for estimation of CO_2_–oil swelling factor. The relative significance of the independent variables including API oil gravity, temperature, pressure, and CO_2_ concentration (mole fraction) on the swelling factor is demonstrated in Fig. 12. As it is clear from the results, the most significant independent parameter is the API degree of the oil samples, temperature holds the second rank, and the CO_2_ concentration exhibits the least impact on the target parameter.

**Fig. 13:**
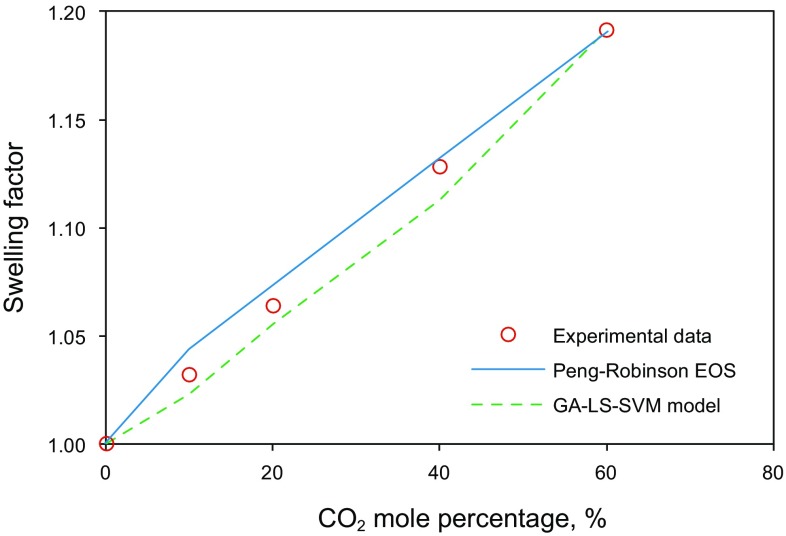
Comparison between the LS-SVM method and Peng–Robinson EOS in terms of performance precision for swelling factor determination

To show the effectiveness of the developed model for a real case, we consider sample AC with the composition reported in Table 5. A swelling test was performed on this sample with different mole fractions of CO_2_. As mentioned previously, one of the methods for swelling factor determination is using EOSs. Thus, the Peng–Robinson EOS as a well-known and robust EOS was utilized to calculate the CO_2_–oil swelling factor of sample AC. Figure 13 displays a comparison between the outputs obtained from the LS-SVM model, Peng–Robinson EOS, and experimental data from a swelling test performed on sample AC. As illustrated in Fig. 13, both LS-SVM and Peng–Robinson EOS predict the CO_2_–oil swelling factor with reasonable accuracy. In this case, the LS-SVM underestimates swelling factor; however, using Peng–Robinson EOS results in overestimating the swelling factor.

**Table 5:** Composition of oil sample AC

Component	Mole percentage, %	Molecular weight
N_2_	0.53	28.014
CO_2_	1.01	44.01
C_1_	45.305	16.043
C_2_	3.9	30.07
C_3_	1.39	44.097
*i*–C_4_	0.63	58.124
*n*–C_4_	0.81	58.124
*i*–C_5_	0.69	72.151
*n*–C_5_	0.41	72.151
C_6_	1.02	86.178
C_7_	4.22	96
C_8_	3.53	107
C_9_	3.5	121
C_10_–C_12_	8.011	145.496
C_13_–C_15_	6.521	186.218
C_16_–C_18_	4.84	227.455
C_19_–C_23_	3.672	283.28
C_24_–C_30_	3.203	370.644
C_31_–C_37_	2.232	469.117
C_38_–C_46_	1.906	579.203
C_47_–C_58_	1.489	722.448
C_59_–C_80_	1.179	940.536

The residual oil saturation, which directly corresponds to the oil recovery factor is inversely proportional to the swelling factor in CO_2_-based EOR processes. Hence, an accurate magnitude of the CO_2_–oil swelling factor increases the precision and reliability of the modeling and simulation studies, which are conducted to capture the main recovery mechanisms and to determine the production performance of CO_2_–EOR strategies for both heavy oil and conventional oil reserves. The present study introduces an accurate and simple-to-use approach to calculate the CO_2_–oil swelling factor, which is an influential parameter throughout CO_2_ injection operations. The precise value of this parameter helps engineers and researchers obtain the residual oil saturation and oil and water relative permeability curves with greater reliability for various oil reservoir development stages (e.g., optimization of operational conditions and economic analysis).

## Conclusions

We used the least-squares support vector machine (LS-SVM) to estimate the CO_2_–oil swelling factor where the extensive experimental data were utilized. The genetic algorithm (GA) was employed to tune the model parameters. The following conclusions based on the research outputs are made:

- The feasibility and performance of the LS-SVM technique with a RBF kernel function were evaluated using the available experimental data on CO_2_–oil swelling factors.
- GA was implemented to determine the optimal extent of the model parameters; namely, regularization factor and variance used in the kernel function which were obtained to be: *γ* = 33.4091 and *σ*^2^ = 0.268829, respectively.
- The hybridized GA-LS-SVM provided excellent results in predicting the CO_2_–oil swelling factor. The performance of the hybrid model was evaluated by *R*^2^ = 0.9953 and MSE = 0.0003, which reveal high accuracy and reliability of the developed model.
- The relative importance of input variables including API gravity of oil, temperature, pressure, and CO_2_ concentration (mole fraction) on the CO_2_–oil swelling factor was investigated using a common statistical approach, ANOVA (analysis of variance). The API gravity of oil, temperature, pressure, and CO_2_ content have the highest to the lowest impact on the objective function in the research study.
- The LS-SVM features high efficiency, excellent generalization, and routine computation methodology, which is suitable for classification and identification of nonlinear cases such as CO_2_–oil systems.

## Appendix: sample calculation

LS-SVM toolbox in MATLAB software is first installed. The next step is dragging and dropping the swelling.mat file in the MATLAB workspace. The following command should be then written in the MATLAB environment to calculate the CO_2_–oil swelling factor.

SF = simlssvm ({trainX′, trainY′, type, gam, sig2, ‘RBF_kernel’, ‘preprocess’}, {alpha, b}, [API T P Solubility (mole faction)])

For example, consider the following data:

API = 29.4°

T = 176 °F

P = 2257 psia

CO_2_ mole fraction = 0.66

SF = simlssvm({trainX′, trainY′, type, gam, sig2, ‘RBF_kernel’, ‘preprocess’},{alpha, b}, [29.4 176 2257 0.66])

The swelling factor forecasted by the LS-SVM model is equal to 0.9327, while the corresponding measured CO_2_–oil swelling factor is 0.9334, implying there is a very good agreement between the predicted and experimental values.
